# Dissociation of Bone Resorption and Formation in Spaceflight and Simulated Microgravity: Potential Role of Myokines and Osteokines?

**DOI:** 10.3390/biomedicines10020342

**Published:** 2022-02-01

**Authors:** Patrick Lau, Laurence Vico, Jörn Rittweger

**Affiliations:** 1 German Aerospace Center (DLR), Institute of Aerospace Medicine, 51147 Cologne, Germany; joern.rittweger@dlr.de; 2 U1059 INSERM–SAINBIOSE (SAnté INgéniérie BIOlogie St-Etienne) Campus Santé Innovation, Université Jean Monnet, F-42270 Saint-Priest-en-Jarez, France; vico@univ-st-etienne.fr; 3 Department of Pediatrics and Adolescent Medicine, University of Cologne, 50937 Cologne, Germany

**Keywords:** bone remodeling, bone formation, bone resorption, unloading, disuse, space medicine

## Abstract

The dissociation of bone formation and resorption is an important physiological process during spaceflight. It also occurs during local skeletal unloading or immobilization, such as in people with neuromuscular disorders or those who are on bed rest. Under these conditions, the physiological systems of the human body are perturbed down to the cellular level. Through the absence of mechanical stimuli, the musculoskeletal system and, predominantly, the postural skeletal muscles are largely affected. Despite in-flight exercise countermeasures, muscle wasting and bone loss occur, which are associated with spaceflight duration. Nevertheless, countermeasures can be effective, especially by preventing muscle wasting to rescue both postural and dynamic as well as muscle performance. Thus far, it is largely unknown how changes in bone microarchitecture evolve over the long term in the absence of a gravity vector and whether bone loss incurred in space or following the return to the Earth fully recovers or partly persists. In this review, we highlight the different mechanisms and factors that regulate the humoral crosstalk between the muscle and the bone. Further we focus on the interplay between currently known myokines and osteokines and their mutual regulation.

## 1. Introduction

Since the beginning of human spaceflight in the early 1960s, space travelers have experienced issues of severe physical deconditioning. As a test bed for long-duration missions, the buildup of the International Space Station (ISS) began in 1999. Since its completion, astronauts have routinely performed missions of varying duration. For the last two decades, mission duration has typically been 6–12 months. These missions have led to new discoveries in biology and medicine. As the planning for deeper space missions to the Moon and beyond have become more concrete and increasingly popular, astronauts will spend prolonged periods of time under conditions of weightlessness. Over the next decade, missions beyond the low Earth orbit as well as lunar exploration and missions deeper into the solar system are among the goals of all mainstream space agencies, such as the National Aeronautics and Space Administration (NASA) and the European Space Agency (ESA). Undoubtedly, human safety and human health preservation during spaceflight remain the topmost priorities considering the goal of further space exploration [1]. Such missions, however, involve inherent physiological challenges that need to be addressed. For decades, it has been known that exposure to microgravity conditions has substantial negative impact on the human body and disrupts normal physiological equilibria. Specifically, the cardiovascular, hematological, immunological, ocular, nervous, and musculoskeletal systems are affected [2,3,4,5,6]. Maintaining the overall integrity of the musculoskeletal system, especially during long-duration spaceflight, is essential for completing missions and maintaining the health of astronauts during and after the mission [7]. In addition, the postflight stressors, especially the ones related to landing and also instances where abrupt re-adaptation to Earth’s gravity is required, must be considered. Therefore, the loss of bone and skeletal muscle mass and strength are major health concerns for astronauts [8,9]. Typically, the muscles most affected are the postural muscles which normally maintain the body’s upright position in a gravity environment [10]. Williams et al. [10] reported a gradual decrease in muscle mass after space flight of 2 weeks, and during long-term space flights, muscle loss may increase without performing countermeasures [11]. Currently, the exercises for high intensity resistance and the aerobic based exercise are considered to be the most preferable strategies for effectively mitigating the effects of microgravity on bone maintenance [12]. Bone and muscle deconditioning are progressive and therefore increase as the mission duration is extended [13]. For a mission to Mars, the estimates for expected bone loss are highly elevated at selected skeletal sites, thereby increasing the risk of developing bone fractures after returning to the Earth, similar to that in the elderly [14]. Considering the mutual dependency of muscles and bones within the musculoskeletal system, it is evident from a mechanical point of view, that muscles generate active forces to which bones react passively. Altogether, this is central to work, locomotion, and posture. Furthermore, bones sustain the body weight and the transmission forces that are generated by muscles to adapt to mechanical loads [15]. Given the mechanosensitive nature of bone tissue, bone adapts its structure and strength to match the localized mechanical loading environment [16,17,18]. Notably, virtually all skeletal muscles constantly work against short levers [19], whereas the largest habitual forces arise from muscle contractions in a gravity environment, such as that of the Earth. In addition, the skeleton acts as a mineral reservoir [20], it holds the hematopoietic bone marrow, and also is critically involved in acid–base homeostasis [21,22]. Therefore, bone tissue is not only mechanically relevant but also interlinked to the endocrine system, with the main effects being on the vital metabolism of calcium, phosphate, and glucose; the fertility; the appetite regulation; and the muscle function [23,24,25,26,27].

To investigate muscle atrophy and bone deconditioning under simulated microgravity conditions on the Earth, tilt bed rest studies with head down at analog −6° are considered the most appropriate ground-based model [28,29,30]. Nevertheless, bed rest is confounded by the presence of gravity and devoid of actual space radiation exposure, such as the effects of galactic radiation characterized by heavy ions and protons which could be chronic and also those caused by occasional solar particle events which mainly involves protons [31]. Chronic exposure to space radiation is also a crucial health risk, especially in case of long duration, deep-space missions about the Moon and Mars [32,33,34]. On the ISS, the astronauts though are protected partially by the Earth’s magnetic field, but they still experience on an average dose equivalent in the range of 180–290 mSv/year [35]. The variation is dependent upon the ISS altitude and the influence of the solar cycle. A better understanding of the interplay between spaceflight factors and human physiology may help address the previously mentioned challenges.

In this review, we discuss the cellular events associated with the process of osteogenic cell differentiation, namely bone formation and resorption, with a brief consideration of other interconnected tissues. In the second part, we discuss musculoskeletal crosstalk and its dissociation under the conditions of modified gravity. In addition, we also highlight the big knowledge gap in space medicine as of now and identify how the translation from microgravity research to applications in space may be possible in the future.

## 2. Osteoblastic and Osteoclastic Cell Function

### 2.1. Origin and Differentiation of Bone Cells

Tissue-specific progenitor cells respond to a broad range of regulatory signals which in turn regulate their proliferation, commitment, and developmental progression and also maintain their structural and functional properties. Bone-forming osteoblasts account for 4–6% of the total resident cells in bone and are originally derived from a common pluripotent mesenchymal progenitor cell [36] which also is the progenitor for chondrocytes, muscle cells, and also the adipocytes [37,38,39]. Bone-forming osteoblasts are typically responsible for bone formation by synthesizing and depositing bone extracellular matrix, which consists of organic (40%) and inorganic compounds (60%) and is primarily composed of type I collagen [40]. Notably, the exact composition of the extracellular matrix differs based on sex, age, and health conditions [40]. The osteoblasts produce the extracellular matrix of bone and control its primary mineralization via a series of commitment and differentiation steps [41]. Moreover, the orientation of collagen fibrils in the bone is also affected by disuse, thereby affecting the mechanical anisotropy of bone tissue [42]. Central to osteogenic differentiation is the balance of bone mass. Thus, osteogenic differentiation requires a balance between osteogenic function and bone resorption, the latter being performed by osteoclasts.

Osteoclasts are believed to be the only cells capable of resorbing bone to create the characteristic scalloped pits and troughs [43]. Osteoclasts originate from the monocyte/macrophage hematopoietic lineage and are multinucleated cells specialized to resorb bone [44,45,46]. During bone remodeling, pre-osteoclasts are recruited to the bone surface, for proliferation and subsequently fusion and differentiation into mature, multinucleated cells [47]. The absence of osteoclast differentiation or dysfunction of its resorption activity causes a bone disease called osteopetrosis, which results in increased bone mass and narrowing of the marrow cavity [48]. Conversely, there is an ongoing debate regarding the possibility that if osteopetrosis occurs first, triggered by osteocyte apoptosis, the lack of a physiological osteocyte/canaliculi syncytium may lead to the withdrawal of signals for osteoclast maturation and migration.

Osteocytes, characterized as mature bone cells have been derived from osteoblasts that have become fully embedded during the process of bone deposition. In the adult skeleton, terminally differentiated and nonproliferating osteocytes are the most common prevalent cell type in bone tissue, representing 90–95% of all bone cells within the matrix [49]. Osteocytes regulate the function of osteoblasts and osteoclasts based on the mechanical and hormonal stimuli received. Osteocytes reside in lacunae and form dendritic processes called canaliculi [50]. Which aid the osteocytes to form networks and interface with other osteocytes, and also other cells on bone surfaces and the bone marrow [51]. Osteocytes secrete sclerostin (SOST), which is a negative regulator of bone mass, and fibroblast growth factor (FGF)-23, a crucial osteocyte-secreted endocrine factor, which is also responsible for phosphate metabolism regulation. In addition, the osteocytes play a major role in mechanotransduction. As potentially mechanosensory cells, they transform the received mechanical strain into efficient chemical signals for stimulation of the effector cells (osteoblasts and osteoclasts). Bone homeostasis and adaptation to normal or diminished skeletal loading are strongly disturbed in mice selectively depleted of osteocytes [52]. Bonewald et al. proposed that osteocyte signaling may be the basis for developing therapeutic approaches for modulating bone mass in diseases, such as osteoporosis, or low-gravity conditions, such as spaceflight [53].

### 2.2. Bone Cellular Activities and Mechano-Coupling

Living cells are constantly exposed to gravity, which impacts cytoskeletal organization and, thereby, the overall cell structure. To adapt to altered gravity, cells convert mechanical input into biochemical signals, activating downstream signaling cascades in a process known as mechanotransduction [54,55]. Under normal physiological conditions, bone structure is maintained by two concurrent processes: bone modeling and remodeling, both of which aid the development and maintenance of the skeletal system. Bone modeling is responsible for shaping bone structures in response to longitudinal growth and overloads. This mechanically induced adaptation of bone requires bone formation to strengthen the structures where needed, which is often accompanied or followed by osteoclastic resorption where it has become superfluous. Although strictly coordinated, these processes occur independently at distinct anatomical locations [56]. Consequently, bone remodeling serves as the renewal of bone tissue by filling up the resorption cavities. This remodeling activity is targeted toward the repair of bone microdamage, thereby preventing fractures resulting from fatigue [57]. In addition, changes in bone shape are associated with aging [58] and responses to mechanical loading. Enhancing bone formation through mechanical stimuli, such as exercise, has several advantages compared with pharmacological interventions because it is body site-specific and autoregulated, involves the entire remodeling cycle, and targets bone-specific genes [59,60]. Thus, bone remodeling occurs in the adult skeleton to maintain mechanical strength and structure. Through this continual process of destruction and synthesis bone maintains its mature structure and regulates normal calcium levels in the body. Notably, remodeling occurs asynchronously in the skeleton in basic multicellular units (BMUs) which are anatomically distinct sites [61]. In trabecular bone, the basic structure of the BMU is located in connection to the bone surface area. In cortical bone, the BMU includes the cutting zones of osteoclasts proceeding through the bone, followed by the differentiating bone forming osteoblasts [62]. Osteoblasts and osteoclasts are the main cells of the BMU. This multicellular unit is comprised of osteocytes and immune cells, which are further discussed below.

Changes in mechanical loading encountered in microgravity markedly influence cell functionality and tissue homeostasis, resulting in altered physiological conditions [63]. A pronounced reduction in bone mineral content associated with microgravity results from an imbalance within the molecular process of bone remodeling and is induced primarily by changes in the morphology, structure, and functionality of these different bone cells (which includes the osteoblasts, osteoclasts, osteocytes, and also the mesenchymal stem cells) [64]. Within the microenvironment of the cellular niche, the differentiation potential, as well as the self-renewal process of stem cells is affected. The lack of gravity and thus the lack of mechanical stimuli leads to an inhibition of osteogenesis with a simultaneous induction of adipogenesis in mesenchymal stem cells [65]. Similarly, osteoblastic differentiation is widely dependent upon signals from osteoclasts and their progenitors. Besides, Cao et al. [66] demonstrates a dysfunction of the homeostasis of the immune system which has been recognized in spaceflight as well as in simulated microgravity conditions on Earth. Since the obtained data sets are scanty, based on limitations in blood sample analysis obtained from space travelers, highly variable responses resulting in dynamic alterations on both hematopoietic stem cells and lineage cells were detected. Due to a decreased expression of Runx2, which contributes to the decreased re-construction capacity of hematopoietic stem cells and lineage cells, the obtained results revealed the osteoblasts to be implicated in the hematopoietic niche of a hindlimb unloaded mice model. Nevertheless, the molecular mechanisms responsible for microgravity induced dynamic alterations of hematopoietic stem cell proliferation are not fully understood. In vitro studies support the concept that simulated microgravity increases osteoclast-dependent bone resorption through increased activity of receptor activator for osteoclastogenesis mediated by the nuclear factor-kappa-B ligand (RANKL) [67], whereas osteoblasts exhibit decreased differentiation under microgravity conditions, as evidenced by decreased expression of collagen type I (COLIA), the bone-specific alkaline phosphatase (bALP), and the osteocalcin (OCN). Ground-based as well as space-related studies also revealed that the transcription factor RUNX2 (cbfa1) is downregulated when preosteoblastic cells are exposed to microgravity, suggesting that cell differentiation is slowed down, but is still ongoing [68]. Notably, in vitro experiments revealed that osteoblasts exposed to significant changes in the mechanical environment exhibited shorter microtubules, thinner cortical actin, thinner stress fibers, smaller and fewer number of focal adhesions. Therefore, the cytoskeleton, which consists of three major components, actin microfilaments, microtubule filaments, and intermediate filaments, has the potential to act as a mechano-sensor [69]. Furthermore, space-flown osteoblasts adopted extended cell shapes, were more disrupted, and often contained fragmented or condensed cell nuclei [70]. Uda et al. [71] reported that genes involved in glucose metabolism and adenosine tri phosphate (ATP) consumption in osteocyte cell lines are the most affected by spaceflight. Conversely, no changes in the regulation of specific gene expression involved in apoptosis and senescence were observed between ground and space conditions, but unloaded ex vivo-cultured osteoblasts exhibited significant upregulation of vascular endothelial growth factor (VEGF), a proangiogenic factor. Thus, one also needs to consider that osteogenesis and angiogenesis are coupled and that the functional management of bone stability may depend on the balance between these two processes [72]. Supporting this view, Grüneboom et al. [73] presented convincing evidence about the network of trans-cortical vessels being the core element for blood circulation in long bones. Further, primary human osteoblasts were exposed for 5 days to simulated microgravity using a desktop random positioning machine. The metabolomic and proteomic analysis of these cells revealed a noticeable dysregulation of mitochondrial homeostasis [74], suggesting that osteoblasts are gravitationally sensitive and functionally impaired from the stress induced by exposure to microgravity.

Since conducting cellular experiments as well as tissue engineering in real microgravity conditions often requires excessive costs and a considerable amount of time, distinct ground-based microgravity simulators were established to mimic mechanical unloading conditions, comparable to those in real microgravity [65]. Simulated microgravity is characterized by the absence of convection, particle sedimentation as well as low shear stress. A number of diverse devices or platforms have been developed including magnetic levitation, two- and three-dimensional clinostats, rotating wall vessels and random positioning machines (RPM). The RPM is characterized by a continuous random change in the orientation relative to the gravity vector and thus an overall averaging of the impact of the gravity vector to zero occurs over time [75]. Detailed information can be found elsewhere [65,76]. Nevertheless, simulation methods have advantages and disadvantages, but properly applied, the results are comparable to those experiments, completed in real microgravity conditions [77].

### 2.3. Coupling and Dissociation of Bone Formation and Resorption

Skeletal homeostasis is maintained by a highly regulated cyclic renewal process. During adult life, this remodeling cycle occurs sequentially on the same cellular surface. The regulation of remodeling is multifactorial, consisting of mechanical factors that emerge from the complex strain patterns associated with structural loads as well as endocrine and paracrine humoral factors [78]. Within these strictly regulated processes, bone resorption requires osteoclast activity followed by bone formation which is mediated by bone-forming osteoblasts. Anatomically, the progression from bone resorption to bone formation, is a unidirectional process called “coupling” and occurs within the BMU. For the maintenance of the bone mass, a posteriori bone formation must match a priori bone resorption, highlighting the importance of resorption–formation coupling [79]. This is consistent with Harold Frost’s proposal that remodeling serves the purpose of substituting old and damaged bone with new bone [61]. In addition to osteoclasts and osteoblasts, bone remodeling requires an under-studied cell type covering >80% of the eroded surface. These quiescent cells are known as bone lining cells; however, their specific role is not completely understood [80]. Evidence suggests that bone lining cells are needed to separate the ionic milieus of bone mineral and the extracellular fluid spaces [81]. Whether these cells have other roles is not yet clear. Therefore, it is not known whether dysfunction of these cells contributes to bone loss in space or in diseases, such as postmenopausal osteoporosis [82,83,84].

The balance between the bone formation and the resorption is more complex than originally thought [85]. This process requires the participation of a number of matrix-derived and membrane-bound factors as well as other cell types [86,87]. With regard to a delay in focal onset of bone formation after resorption is complete, Parfitt concludes that although defective coupling occurs in many osteoporotic patients, it makes only a minor contribution to bone loss [62]. Regarding weightlessness conditions, zero gravity as well as simulated microgravity on Earth adversely affect the bone remodeling cycle at all phases. Under these conditions, the maturation and resorptive activities of osteoclasts increase, whereas their differentiation, maturation, and bone-forming abilities decrease. This is explained in more detail below. As Sims and Martin concisely stated in their review [79], that caution is required, since for other different times in life, though the balance may be negative (bone mass is reduced) or positive (bone mass is gained), but this does not imply that the coupling is interrupted (though the term “uncoupling” is often used). Bone formation still follows resorption, but there is an imbalance between these activities of the bone cells.

Functional loading of the skeleton is critical for achieving and maintaining adequate muscle and bone quantity and quality throughout life. Fortunately, the adaptive response of bone towards the mechanical stimuli indicates its ability to decipher the mechanical information, such as strain, and translate it into biological instructions. Therefore, consistent physical activity affects the bone mass positively whereas the physical inactivity, such as during spaceflight, induces bone loss. In addition, physical exercise is an effective countermeasure to protect against such experimental induced bone loss. However, the mechanotransduction of bone is far from being well understood [88]. Multiple molecular mechanisms, which act together, have been proposed to explain the mechanotransduction process in bone cells. Osteocytes are finely tuned sensors of mechanical stimulation which are responsible for coordinating the coupling of bone resorption mediated by osteoclasts to bone formation mediated by osteoblasts [49]. These processes involve multiple local, and paracrine signals diverted from osteoclasts, osteoblasts as well as bone matrix which acts on cells of the bone remodeling compartment. In blunt contrast, to the negative impact of weightlessness on osteoblasts [55], in which the cytoskeleton collapses from the failure of microtubules and actin filaments, for the osteoclasts their ability to resorb bone in microgravity is still maintained [70]. These changes along with the diminished force of muscular contraction on bone, are responsible at least in part, for the space-related loss of bone mass and also the dissociation of bone formation and resorption under simulated microgravity conditions [89].

Osteoclast formation may be initiated by multiple factors released locally from various cell types, such as the mature osteoblasts, osteocytes, the lining cells, and the preosteoblasts [90]. Understanding the specific mechanisms by which osteoclasts communicate with nearby osteoblasts during bone remodeling is central because bone mass depends on the bone remodeling balance. However, it should be noted that it is not yet clear if the osteoclast products influence the osteoblast lineage, at the various stages of differentiation [91]. Compared with macrophages, osteoclasts express high levels of the known anabolic molecules—bone morphogenic protein (BMP)-6 and Wnt10b—suggesting their function as coupling factors [92]. The first proposed coupling factors identified from cell culture studies, were transforming growth factor β (TGF-β) (which is stored in large amounts in the bone matrix and released and activated by osteoclasts during bone resorption) and insulin-like growth factor 1 (IGF)-1 [85]. Subsequently, it was suggested that TGF-β may also act directly on the osteoclastic lineage to cause induction of coupling factors expression [93]. Resorption-derived IGF-1 promotes differentiation of osteoblast by recruiting mesenchymal stem cells and activating mammalian target of rapamycin (mTOR) [94]. Recently, Durdan et al. [95] stated that TGF-β in the matrix polarizes the osteoclast lineage cells towards a “coupling” phenotype, which leads to the simultaneous expression of bone anabolic proteins, which further get concentrated within the bone remodeling compartment. Osteocytes also increased RANKL expression, which may contribute to increased osteoclastic bone resorption. In addition to soluble factors, cell–cell interactions between osteoclasts and mesenchymal lineage cells through membrane factors have been proposed as mechanisms for coupling resorption to formation [95]. Furthermore, the possibility of transport of vesicular coupling factors has been proposed [91]. Although, the most recent observation of membrane interaction coupling osteoclasts and osteoblasts is that of the reverse signaling through RANK and RANKL partners [96].

**Figure 1:**
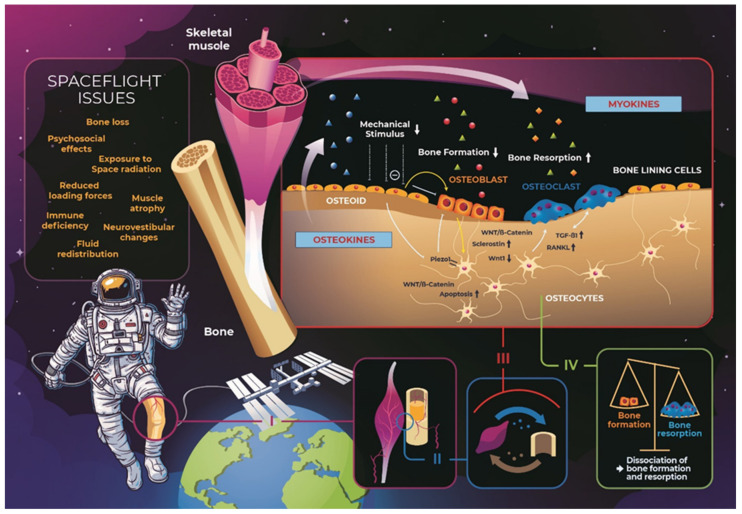
Schematic illustration of the most important spaceflight health issues experienced by space travelers. The microgravity environment exerts a multitude of human health hazards (e.g., psychological effects, exposure to space radiation, immune deficiency and microgravity). Focusing on the musculoskeletal system, weightlessness conditions promote muscle atrophy as well as bone loss (I). Considering their spatially distance, it is most likely, that muscle and bone are metabolically interconnected and highly vascularized (II). Osteocytes sense external mechanical signals and transduce them into internal biochemical signals. Therefore, they are realized as the principal regulators of bone mechanosensation and mechanotransduction. Mechanical loading or unloading increases the osteocyte membrane tension which further induces the opening of PIEZO1 channels. Specifically, mechanically activated nonselective Ca^2+^-permeable cation channels of the PIEZO family (PIEZO1 and PIEZO2) are recognized as the most important mediators of mechanotransduction. SOST, a protein secreted by osteocytes is a member of the Dickkopf family, and also a potent suppressor of canonical Wnt-β-catenin signaling pathway via Lrp5/6, negatively regulates bone formation. Under weightlessness conditions, upregulation of TGF-β1 as well as RANKL has been considered as an important indicator of osteoclast activation and differentiation (III). Myokines and osteokines are released by skeletal muscle and bone tissue into the blood circulation. Microgravity therefore triggers the dissociation of bone formation and resorption (IV).

It has become increasingly obvious that the remodeling process is synergistically coordinated via multiple mechanisms, soluble and membrane factors, as well as effects on the bone matrix [95]. Several different molecular pathways directly mediate the skeletal unloading. The loading environment changes are primarily perceived via complex, and exquisite regulatory pathways between surrounded cells and the mechano-sensitive osteocytes, that are deeply embedded in the mineralized bone matrix (Figure 1). Specifically, mechanically activated nonselective Ca^2+^-permeable cation channels of the PIEZO family (PIEZO1 and PIEZO2) are recognized as the most important mediators of mechanotransduction. Thus, it has been hypothesized that PIEZO1 functions as a “mechanostat,” which directly senses the mechanical loading which aids to coordinate the osteoblast–osteoclast crosstalk and regulating the subsequent responsive gene expression [97].

SOST, an osteocyte-secreted protein and a member of the Dickkopf family is a potent suppressor of the canonical Wnt-β-catenin signaling pathway. It binds to the Lrp5/6 protein which is a crucial negative regulator of bone formation. SOST inhibits osteoblast activity thereby playing a vital role in controlling the response to mechanical loading [98,99,100]. SOST also inhibits bone morphogenic protein-related bone formation [101] and is increased in healthy adult men undergoing head-down tilted bed rest [102,103]. The secretion of SOST upregulates the bone resorption by decreasing osteoblast derived osteoprotegerin (OPG) production. In partial contrast to this finding, Belavý et al. [104] reported increased SOST expression with bed rest; however, the effectiveness of resistive vibration to prevent bone loss during bed rest was not reflected by the expected suppression in SOST elevation. Similarly, while using the advanced resistive exercise training device on the space station, which enables astronauts to perform high intensity workouts, no statistical significant differences in the serum SOST levels, were demonstrated [105]. Nevertheless, anti-SOST antibodies, such as romosozumab, blosozumab, and BPS804, represent a promising group of anabolic agents that prevent the inhibitory effect of osteocyte-derived SOST on osteoblastic Wnt signaling and thus increase bone formation [106,107]. Therefore, Wnt signaling is recognized as one of the most important pathways with respect to bone response to mechanical loading [108]. Bone gla-protein or OCN is involved in the crosstalk between bone and muscle and is an indicator of bone metabolism during spaceflight. OCN is produced by osteoblasts and has been widely used as a bone formation marker [109]. The carboxylation state of BGP is respective to alterations in vitamin K status [110]. The serum concentrations of undercarboxylated OCN are increased in elderly women which could be due to changes in bone quality and not the mineral content [111]. Caillot-Augusseau et al. [112] stressed that microgravity induces an early uncoupling of bone remodeling and reported an upsurge in undercarboxylated BGP during both, the short-term and the long-term spaceflight, which is suggestive of impaired metabolism of vitamin K. As previously noted, muscle and bone interact anatomically and biochemically. Therefore, changes in mechanical stress resulting from immobilization or lack of gravity influence both bone and muscle. Both tissues communicate through common pathways and coordinate with one another via paracrine signals known as myokines and osteokines [113]. Interestingly, the muscle loss has been reported to recover approximately 6 months faster than that of bone loss in astronauts [114]. Several lines of evidence have indicated previously that low magnitude mechanical signals act anabolic to bone and muscle [115]. There are several factors that negatively affect bone and muscle, including physical activity, hormones, the nutritional state, post-inflammatory cytokines and also atherosclerosis [116].

## 3. Musculoskeletal Crosstalk

### 3.1. Bone and Muscle Loss in Microgravity

Under microgravity conditions, unloading primarily affects weight-bearing skeletal regions that help to move the body’s mass under normal gravity conditions [117,118,119]. Given that bone tissue is mechanosensitive, bone and skeletal muscles are highly responsive to changes in their mechanical loading environment (Figure 1). In addition, as already mentioned, bone and muscle also act as endocrine organs [120]. Notably, weightlessness-induced bone loss may be more severe than osteopenia on the Earth, and prolonged exposure to unloading conditions significantly increases the risk of osteoporosis and bone fractures [121]. Bone loss must be expected to develop instantaneously once arrived in space. During the first days after the start of a mission, also a 60–70% rise in levels of urinary and fecal calcium has been reported, which continues throughout the duration of the mission [122]. Elevated calcium levels in the blood lead to a decrease in parathyroid hormone (PTH) secretion [123]. PTH inhibits the secretion of OPG, allowing for preferential differentiation into osteoclasts. This pronounced inhibition leads to a reduction in vitamin D production at the intestinal level, leading to decreased kidney Ca^2+^ reabsorption and hypercalciuria, consequently increasing the risk of kidney stone formation [124]. Notably, there are large differences in bone density loss after a 6-month mission, typically accounting for 8–12% [125]. The loss of bone mineral density (BMD) at sides in femoral neck, spine and trochanter, and pelvis is equivalent for 1.0–1.6% every month. The loss of bone mass in the whole body and legs, which are rich in cortical bone, is approximately 0.3–0.4% per month [118,126,127,128]. Stavnichuk et al. [129] recently reported that it is difficult to produce data on different skeletal region changes and also on changes in the temporal kinetics of bone loss. Moreover, the alterations in bone density levels are dependent, on the site and bone gains in the cervical vertebrae and the skull as well as the progressive bone loss in the lumbar spine, pelvis, and lower limbs are most common [130].

In addition to bone, the exposure to actual or simulated microgravity effects skeletal muscle similarly, whereas the postural muscles are the most affected. Lack of the stimulus of the mechanical loading on the musculoskeletal system results in biochemical and structural changes, atrophy, and muscle wasting, which further puts the astronauts at risk for injuries when in spaceflight and also upon normal gravitational reloading [1]. The adaptation of skeletal muscle occurs largely in response to changes in the mechanical environment. After a 2-week space flight, the muscle mass is reduced by up to 20% [131]. On longer missions of about 3–6 months, there is an average skeletal muscle wasting loss of up to 30% [10]. Rittweger et al. [132] presented convincing evidence that studies on two astronauts demonstrated decrements in the aerobic metabolism in muscles and also in phosphate high energy transfer. Additional factors that contribute to muscular loss include suboptimal nutrition and stress which can also influence bone [122].

Gross muscle atrophy is paired with a reduction in the size, not the number, of muscle fibers. Protein synthesis in muscle fibers is decreased, whereas protein degradation is raised. In the new in-flight equilibrium state, protein synthesis is decreased by 15% compared with preflight conditions, and fiber cross-sectional areas are reduced by an amount of 20–50%. To understand the impact of spaceflight on the musculoskeletal system, bed rest studies have been used as a surrogate model for spaceflight-induced bone loss [133]. Bed rest-induced metabolic changes in BMD, calcium excretion, calcium balance, and bone markers are similar to (although quantitatively somewhat less than) those documented during spaceflight [126]. More recent data presented that bone loss during bed rest is approximately half of that observed in space [134].

### 3.2. Role of Myokines and Osteokines in the Crosstalk between Bone and Muscle

The profound relationship existing between the skeletal muscle and bone has been highlighted in the last few years, with a steady increase in the number of publications [135]. Originally, the musculoskeletal system was characterized as a multipart organ system that involves numerous structural components (e.g., bones skeletal muscles, tendons, ligaments, connective tissues, vasculature, the joints and the nervous system). In particular, bone primarily plays a role in mechanical support and protection of the internal organs, whereas muscle generates force and produces movement. In addition to their functional, developmental, and metabolic liaison, the muscle and bone tissues, both coordinate via mechanical interactions and through a fine-tuned system of molecules and soluble factors, acting through, the paracrine, endocrine and the autocrine mechanisms [136,137]. The fact that after exercise, specific cytokines specifically produced in muscle, reach high concentrations in the blood-stream strongly indicates that myokines are released into the circulation, directly [138,139,140]. More recently, the relationships between the muscle and the bone are considered a novel research field and are correlated with several other organ systems interactions [141].

**Figure 2:**
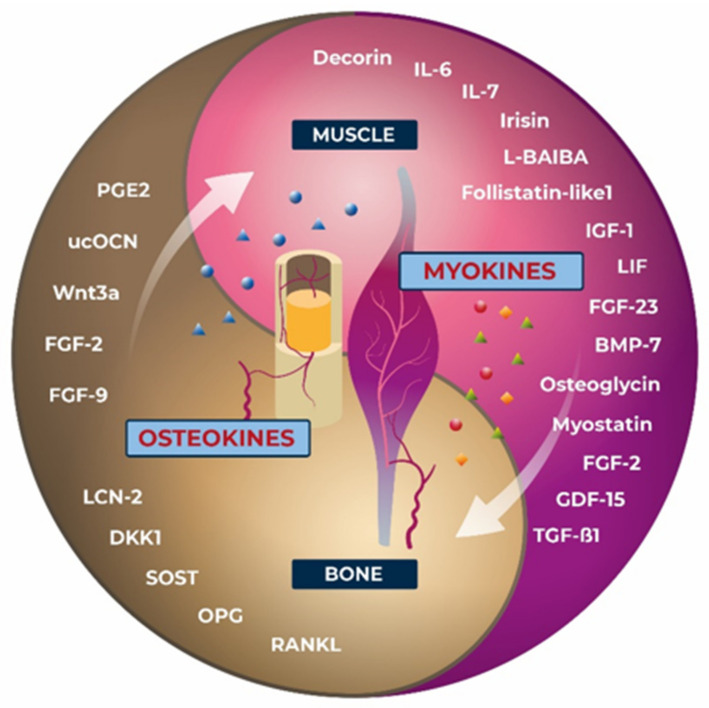
Myokines and osteokines produced and secreted by the skeletal muscle tissues and the bone tissues. Myokines like Irisin, IGF-1and L-BAIBA, LIF and BMP-7 positively alter the bone tissue by upregulating the activity of osteoblasts. In parallel myokines like myostatin, IL-6, IL-7 and FGF-23 stimulate bone resorption and therefore negatively regulate bone mass. Conversely, several bone cell-derived factors circulating in the periphery named osteokines influence local and systemic metabolism. They directly stimulate myogenesis and affect muscle function. Osteokines such as ucOCN, derived from bone affect skeletal muscle positively, which in turn improves muscle functions during exercise. Others such as SOST, DKK1 and RANKL effect the muscle function negatively which in turn reduces muscle function and strength.

As bone and muscle cells are originally derived from the same mesenchymal stem cell precursors, differentiation-related gene reprogramming between bone and muscle tissue is highly likely, particularly during the early growth and development. Several studies have suggested that the indian hedgehog pathway (IHH) and FGF-2 play a major role in the interactions between muscle and bone during development [142]. During embryogenesis, muscle grows more rapidly than bone, suggesting that during growth may enhance subsequent bone accrual [143]. The functional activities of the skeletal muscle and bone reciprocally affect each other (and also other tissues and organs) via the crosstalk mechanisms based on molecular mediators of development and aging. These crosstalk’s includes several molecular, biochemical, and genetic coupling that we began to comprehend only recently [135,136]. In response to muscular contraction, skeletal muscles secrete several peptides, growth factors, and cytokines as well as soluble molecules known as myokines, which enter the blood circulation, most probably by diffusion through the periosteum, and affect bone tissue [144]. Muscle-derived myokines are taken up by osteoblasts, osteocytes, and osteoclasts, thereby regulating bone resorption and formation (Figure 2). More than 3000 myokines have been identified so far and these are extensively reviewed elsewhere [145,146,147,148]. Moreover, the number of newly discovered myokines is increasing [149]. It is noteworthy that the majority of these myokines are still not sufficiently characterized; therefore, only selected myokines with known effects on bone are discussed here (Table 1). One of the first identified muscle-derived soluble factors was myostatin, also known as growth differentiation factor 8, previously. Being a member of the TGF-β superfamily, myostatin regulates the skeletal muscles—positively and negatively, it not only suppresses skeletal muscle mass and development but also negatively regulates bone mass [150]. Bone matrix decorin has been identified as a myokine, and it is regulated by exercise and acts as an antagonist to myostatin. Through binding to TGF-β, an inhibitory effect on the proliferation of osteoblastic cells is enhanced [151]. TGF-β as well as BMPs are important regulators of bone and muscle formation and homeostasis [152]. TGF-β1 is released and activated due to the activity of bone-resorbing osteoclasts. Irisin (FNDC5) produced in skeletal muscle after exercise has been found to be protective towards insulin resistance and cardiovascular disease [153]. In bone, irisin upregulates the osteoblast differentiation via the canonical Wnt-β-catenin, p38MAPK, and ERK signaling pathways. It suppresses osteoclast differentiation by suppressing the RANKL/NFATc1 pathway [147]. These findings indicate that irisin could be a potential marker for the assessment of muscle/bone disorders, which may also be relevant to physiological changes during spaceflight. In addition, after exercise numerous interleukins and chemokines are released from the muscles. IL-6 affects bone metabolism and stimulates the resorption of bone and, therefore, could be linked to pathogenesis of postmenopausal osteoporosis [154]. However, a controversy exists because IL-6 activity may depend on concentration, timing, or duration of the signal. Leukemia inhibitory factor (LIF), member of the IL-6 cytokine family, is an exercise-induced myokine. Through autocrine and paracrine signaling, LIF induces skeletal muscle hypertrophy and regeneration [155]. It stimulates bone formation in vivo [138] and exerts multiple biological functions [148]. IL-7 affects both osteoblasts and osteoclasts [156]. One of the most important mediators of muscle and bone growth remains IGF-1 [157], which is produced by skeletal muscles during physical activity [158]. Specifically, the GH/IGF1 axis delivers the crucial required stimulus for regulating the bone growth, by triggering the differentiation of osteoblasts [159]. Consistent with current findings, Zhao et al. [160] correlated a decline in GH and IGF-1 secretion with BMD loss in postmenopausal woman. Overall, IGF-1 may represent an interesting candidate for musculoskeletal research during weightlessness conditions. In unloading models like in rodents [161,162,163] and humans [164], increased circulating IGF-1 levels were evidenced, which correlated with a decline in bone formation. Such findings are suggestive that unloading causes to induce a development of resistance to this growth factor. In rats submitted to hindlimb unloading [165] even the failure of the unloaded bone to grow in response to exogenous IGF-1 was detected.

β-aminoisobutyric acid (L-BAIBA) is another relevant protein, being a novel muscle-derived molecule, which exerts both direct and indirect anabolic effects on bone cells. It is produced by skeletal muscles during exercise and preserves muscle strength [187].

Myokines have provided a new paradigm and a conceptual basis for understanding the crosstalk between muscles and other organs or tissues. Conversely, more recent reports have provided evidence that several bone cell-derived factors known as osteokines, which influence local and systemic metabolism, circulate in the periphery, directly stimulating myogenesis and affecting muscle function (Table 2). The most widely studied osteokines are derived from osteoblasts, osteocytes, and their precursor cells [204]. Among the first described, two factors secreted by osteocytes in response to shear stress are prostaglandin E2 (PGE2), the secretion of which is further enhanced under loading conditions, and Wnt family member 3a (Wnt3a) [205]. Sassoli et al. reported that mesenchymal stem cells of the bone marrow stimulate myoblast proliferation via VEGF secreted by mesenchymal stromal cells, suggesting that bone mesenchymal cells affect the muscle cells. As IGF-1, MGF, myostatin, VEGF, and hepatocyte growth factor (HGF) are produced in bone cells, they may act as anabolic and metabolic factors regulating muscle mass [206]. Moreover, RANKL has been considered an important indicator of osteoclast differentiation [207]. RANKL and its receptor RANK are part of the upstream signaling of the nuclear factor-κB (NF-κB) pathway, the key transcription factor inducing proinflammatory gene expression. Notably, the receptor for RANKL is further expressed in skeletal muscles. Its activation inhibits myogenic differentiation, resulting in skeletal muscle dysfunction [208]. OPG is a soluble receptor of RANKL that prevents it from binding to RANK, leading to the inhibition of osteoclast production. FGF-23 is recognized as the first hormone-like osteokine secreted by osteocytes. In coordination with PTH, it jointly regulates phosphate metabolism [209]. As previously described, OCN is the most abundant noncollagen protein secreted in a carboxylated form solely in osteoblasts. Because of its low pH and decarboxylation, it may be released into the blood circulation [181]. OCN also stimulates the synthesis of IL-6 [210]. The prevalent form of OCN in serum is undercarboxylated. Bioactive ucOCN and the circulating levels of ucOCN increase after exercise [211]. SOST, a glycoprotein and osteokine released by osteocytes, may be involved in muscle mass modulation. Von Maltzahn et al. [212] reported that the Wnt signaling pathway plays an essential role in the differentiation of muscle stem cells. Moreover, recent research has found that skeletal muscle is a new source of SOST [213]. Lipocalin-2 (LCN-2) is the most recently identified osteokine. LCN-2 is secreted by a spectrum of cells which include the neutrophils, adipocytes, macrophages, liver and kidney [214]. Recently, it has been reported in mice, that LCN-2 is produced ten-fold more in bone than in white fat [27]. Further, a role for LCN-2 in energy metabolism has been proposed. Rucci et al. [215] demonstrated that LCN-2 might be a mechanoresponding gene, which may correlate with poor osteoblast activity [216]. LCN-2 is upregulated in human and animal models of reduced mechanical forces [215]. In addition, it is found to cross the blood–brain barrier in order to interact with melanocortin 4 receptor (MC4R) in the hypothalamus and subsequently inhibit the appetite post binding [27]. This further strengthen the idea that bone is acting as an endocrine organ. He et al. [204] further stressed the influence of extracellular vesicles (EVs) on cell–cell communication, considering that they may be secreted from bone to muscle and vice versa. Several microRNAs may also get transformed in the bone cell-derived EVs, which can affect the bone metabolism and also cause regulation of the muscle metabolism under altered gravity conditions. In addition, small molecules, such as ATP and nitric oxide (NO), may act as osteokines owing to their effects on bone [23]. Myokines, which may increase or decrease in the circulation after exercise or disuse, and more recently, osteokines, which affect one another, may be useful biomarkers in the future, especially with respect to the physiological and metabolic response to spaceflight or simulated microgravity conditions.

## 4. The Bone Cell Differentiation Paradox: An Issue for Bone Recovery?

Although there is extensive evidence of bone loss in astronauts during long-term spaceflight, there are only a few datasets available on bone recovery once returned to Earth. Skeletal reloading studies on animals and humans, after spaceflight have demonstrated that even after recovery periods longer than the period of unloading, there is incomplete recovery of bone mass and architectural parameters [119,227,228]. Thus, bone loss is expected to stop and be reversed in real and simulated weightlessness after immobilization has ended. Nonetheless, bone loss continues after reambulation [229]. Indeed, post-reambulatory bone loss has consistently been observed in ground-based analog studies [230,231,232,233]. Surprisingly, there has also been post re-entry bone loss observed in mice that flew in space for one month [234]. With respect to the cellular dynamics of bone adaptive responses, osteoclastic bone resorption is moderately elevated within a few days of immobilization. However, the full osteoclastic resorption response is protracted by approximately 1 week [233,235,236]. Conversely, it requires 1 week after reambulation for osteoblastic formation to reach the full response level. The same time delays have recently been established, not only for biochemical bone markers but also for calcium fluxes (i.e., the element measured by the radiological assessment of bone) [237]. The evidence that post-reambulatory bone loss is associated with bone cellular dynamics is fairly consistent. The overall mechanism cannot be determined by the modulation of osteoclastic and osteoblastic activity alone. There are time delays involved in the recruitment of osteoclast and osteoblast precursor cells and the differentiation of these cells [238]. During a normal remodeling cycle, osteoclastic resorption takes ~42 days, followed by a reversal phase, which requires ~9 days, before bone formation, which takes ~145 days [239]. Furthermore, the coupling signals between osteoclasts and osteoblasts are not yet fully understood [240]. Osteoclasts may need to complete their resorption cycle before they can signal to the osteoblasts to start forming bone, which causes further delay. Therefore, the abovementioned rapid increase in bone resorption markers indicates an almost immediate alteration of osteoclastic activity in response to changes in the mechanical environment of the bone. The second “boost” in bone resorption would result from delays in osteoclastic differentiation and potentially migration and homing. It may further depend on signaling between myokines and osteokines. Evidence for the latter has recently been provided in a study on the CD200/CD200R system, despite these observations being observed during bed rest [241]. In the early detection of osteoporosis in space, i.e., the “EDOS-1” study, 13 cosmonauts were assessed before and for a time period of 12 months after a 4–6-month sojourn in space [118]. Bone remodeling markers, uncoupled in favor of bone resorption at landing, returned to preflight values within 6 months (i.e., the duration of a bone remodeling cycle in humans) [239]. Between 6 and 12 months postflight, they declined further to a level lower than the preflight values. Interestingly, this diminished turnover of bone was coinciding with the thinning of cortical tissues and also a trabecular loss in radius, which was spared at landing. The underlying mechanisms are not yet understood, although an osteocyte origin is suspected. With this information, the ESA designed “EDOS-2” to confirm that bone loss after re-entry also occurs in astronauts, analogous to post-reambulatory bone loss after bed rest, and to characterize the extent to which bone marker level deteriorate and the bone structure trigger or bone fragility is exacerbated in a better possible way. Biochemical markers of bone resorption, serum cross-linked C-telopeptide of type I collagen (CTX) and bone formation, N-terminal propeptide of type I procollagen (P1NP) as well as SOST were assessed. The overall aim is to analyze 15 astronauts. These results may help to evaluate post-reambulatory bone loss and lack of full recovery after short- and long-term spaceflight.

## 5. Conclusions and Perspectives

Exposure to spaceflight-related health hazards affects the whole body, mostly in a way that is disruptive to normal physiological equilibria, including the cardiovascular, hematological, immunological, ocular, neurological, and musculoskeletal systems. Although many studies have increased our understanding of these physiological changes within the last few years, there is much to discover regarding real and simulated microgravity-induced adaptation processes. For instance, although it is understood that when astronauts return from extended space flight, they must remain active to maintain their skeletal strength, uncertainty continues to exist as to whether bone is as strong after spaceflight as it was preflight, whether bone fully recovers, and whether nutrition and exercise can be optimized during and after spaceflight [2,118]. Therefore, bone loss remains a key medical concern when sending humans to space, in particular, for the already-planned deep-space missions. Bone loss appears to continue even after reambulation, which has been demonstrated in bed rest studies [88,231]. In the context of space-related bone research, post-reambulatory bone loss involves several unanswered questions: Why is bone loss protracted in some cases and not in others? What are the physiological mechanisms underlying bone cellular adaptation dynamics? Medication to protect from bone loss continues to be a realistic option, at least in the initial phase of deep-space travel. Solving this challenge and understanding the integral cellular mechanisms would be highly informative for the selection of such medications and the development of countermeasures. In addition, spaceflight involves enormous challenges that are difficult to replicate on the Earth, in particular, with respect to its impact on bone. On the Earth, the number of osteoporotic fractures remains high, with often catastrophic consequences for patients. Therefore, a mechanistic understanding of bone loss in space as well as on the Earth as well as its underlying factors will be a significant step for medical science. This will directly and indirectly contribute to the improved management of bone maintenance for astronauts. Stavnichuk et al. [129] recently demonstrated the feasibility of exploratory studies based on comprehensive review of literature towards developing concepts for mechanistic understanding of bone density changes in astronauts. This is highly essential in order to plan and design subsequent successful countermeasures. Additionally, open-access platforms, such as the NASA GeneLab database (Retrieved, 1/25/2022, from https://genelab.nasa.gov, accessed on 25 January 2022), accumulate, curate, and provide access to the genomic, transcriptomic, proteomic, and metabolomic (“omics”) data from biospecimens flown in space or exposed to simulated space stressors. A recent example is the NASA Twin study, which has provided a detailed look at multi-omic measures after spaceflight and revealed the changes that occur [242]. In addition, machine learning algorithms and artificial intelligence can take full advantage of the usage of this data, particularly for modeling and extrapolation to human health risks [243]. The availability of astronauts is a limiting factor and interindividual variation, in which participants display markedly different responses to a standard intervention [244], is recognized as a key characteristic of spaceflight-induced adaptations. Therefore, precision or personalized medicine is the key to further space exploration. The development of new genetic, biological, and bioinformatics-based approaches and their application is important. Minimally invasive measurements using biofluids (microRNA transcription) may also play a crucial role in the elucidation and our understanding of the molecular mechanisms of microRNA regulation in bone remodeling and its therapeutic implications for osteoporosis [245]. To prepare the way for future long-term space flight scenarios, it is necessary to gain deeper insight into how exposure to microgravity affects bone formation and resorption under real gravity conditions. Furthermore, simulated microgravity experiments may contribute in a substantial manner.

## Figures and Tables

**Table 1:** Selected myokines with proven functions in humans and their effects on bone.

Marker	Abb.	Action	Ref.
Brain-derived neurotrophic factor	BDNF	Regulates VEGF secretion by osteoblasts.	[166]
Bone matrix decorin	DCN	Binds to TGFβ and enhances its inhibitory effect on the proliferation of osteoblastic cells, is regulated by exercise, and acts as an antagonist to myostatin.	[151,167]
Bone morphogenic protein 7	BMP-7	Important factor in bone formation and skeletal muscle mass maintenance. Induces osteoblastic cell differentiation of C_2_C_12_ cells.	[168,169]
Fibroblast growth factor 2	FGF-2	Localized to muscle–bone interface in vivo, SOST signaling inhibitor.	[146,170]
Fibroblast growth factor 21	FGF-21	Mediator of glucose uptake in skeletal muscle, leads to bone resorption.	[171]
Follistatin-like 1	Fsl-1	Negative regulator of muscle growth.	[172]
Growth differentiation factor 15	GDF-15	Secreted from skeletal muscle in response to mitochondrial stress.	[173]
Insulin-like growth factor 1	IGF-1	Secreted from cultured myotubes in vitro, stimulates bone formation both in vitro and in vivo. Receptors are abundantly localized to the periosteum at the muscle–bone interface.	[174]
Insulin-like growth factor-1Ea	IGF-1Ea	Expression of the full propeptide protects against age-related loss of muscle mass and strength.	[175]
Interleukin 15	IL-15	Supports osteoblastic matrix formation, potent proliferator of innate immune cells.	[176]
Interleukin 6	IL-6	Increases osteoclast activity, proinflammatory. Increases osteoblast activity. Effects may depend on concentration, timing, and/or duration of the signal.	[177,178]
Interleukin 7	IL-7	Promotes osteoclastogenesis and inflammatory responses and inhibits bone formation.	[179,180]
Interleukin 8	IL-8	Positive effects on muscular angiogenesis.	[181]
Irisin (fibronectin type III domain containing 5)	FNDC5	Anabolic effect on bone, improves osteoblastogenesis, improves bone mass in animal models.	[182,183,184,185,186]
β-aminoisobutyric acid	L-BAIBA	Prevents osteocyte cell death, preserves bone and muscle, blood levels increase in response to constant exercise, and regulates bone and skeletal muscle loss due to aging.	[187,188]
Leukemia inhibitory factor	LIF	Stimulates bone formation in vivo.	[189]
Matrix metallopeptidase 2	MMP-2	Involved in bone formation and metabolism.	[190,191]
Musclin/Osteocrin	OSTN	Exercise-induced myokine and is produced by osteoblasts. Specific ligand for natriuretic peptide clearance receptor which modulates bone growth.	[192,193,194,195,196]
Myostatin (growth/differentiation factor-8)	GDF-8	Negative regulator of muscle mass and inhibits osteoblastic differentiation. Exercise reduces its secretion. Promotes osteoclastogenesis induced by RANKL in vitro.	[136,143,197,198]
Osteoglycin	OGN	Inhibits myoblast migration during myogenesis.	[199,200]
Osteonectin (secreted protein, acidic, rich in cysteine)	SPARC	Elevated levels in muscle and plasma of mice and humans post-exercise. Exercise reported to induce osteonectin secretion from the muscle tissue.	[201,202,203]
Transforming growth factor beta 1	TGF-β1	Stimulates matrix protein production by osteoblasts. Released and activated due to osteoclasts during bone resorption.	[147]

**Table 2:** Selected osteokines with proven functions in humans and their effects on muscle.

Marker	Abb.	Action	Ref.
Undercarboxylated osteocalcin	ucOCN	Positive effects on the muscle mass and associated functions. Vital for adaptation to exercise. Insulin-dependent increase in glucose uptake in mice.	[217,218]
Dickkopf 1	DKK1	Catabolic osteokine that downregulates bone formation through the inhibition of the Wnt pathway. Expressed by osteocytes and osteoblasts.	[219]
Sclerostin	SOST	Suppresses Wnt3a-mediated crosstalk between MLO-Y4 osteocytes and muscle cells C_2_C_12_ by regulating the Wnt/β-catenin pathway. Inhibition restores muscle function in cancer-induced muscle weakness. Muscle-derived SOST works synergistically with bone-derived SOST to strengthen the negative regulatory mechanisms of osteogenesis.	[213,220,221]
Insulin-like growth factor 1	IGF-1	Bone formation stimulation found in vitro and in vivo. Important myokine for bone.	[146,174]
Fibroblast growth factor 9	FGF-9	Expressed in bone. FGF-9 mRNA expression is highly enriched in osteocytes.	[222,223]
Fibroblast growth factor 23	FGF-23	Mainly produced in osteocytes. Crucial regulator of phosphate and calcium metabolism via multiple organs.	[224]
Osteoprotegerin	OPG	Main regulator for osteoclast differentiation and also the bone remodeling. Novel protector of muscle integrity.	[225]
Receptor activator of NF-κB ligand	RANKL	Inhibits muscle mass and function.	[181]
Wnt family member 3a	Wnt3a	Wnt3a accelerates C_2_C_12_ differentiation.	[220]
Prostaglandin E2	PGE2	Mimics specific effects of the osteocyte-secreted factors on the process of myogenesis and also the muscle function.	[205]
Fibroblast growth factor 2	FGF-2	Involved in normal skeletal growth.	[226]
Lipocalin-2	LCN-2	Mechanoresponding gene, which may correlate with poor osteoblast activity	[215]

## Data Availability

Not applicable.
